# HIV-1 subtype and viral tropism determination for evaluating antiretroviral therapy options: an analysis of archived Kenyan blood samples

**DOI:** 10.1186/1471-2334-9-215

**Published:** 2009-12-30

**Authors:** Raphael W Lihana, Samoel A Khamadi, Raphael M Lwembe, Joyceline G Kinyua, Joseph K Muriuki, Nancy J Lagat, Fredrick A Okoth, Ernest P Makokha, Elijah M Songok

**Affiliations:** 1 Centre for Virus Research, Kenya Medical Research Institute, Nairobi, Kenya; 2 Department of Medical Microbiology, University of Manitoba, Winnipeg, Manitoba, Canada

## Abstract

**Background:**

Infection with HIV-1 is characterized by genetic diversity such that specific viral subtypes are predominant in specific geographical areas. The genetic variation in HIV-1 *pol *and *env *genes is responsible for rapid development of resistance to current drugs. This variation has influenced disease progression among the infected and necessitated the search for alternative drugs with novel targets. Though successfully used in developed countries, these novel drugs are still limited in resource-poor countries. The aim of this study was to determine HIV-1 subtypes, recombination, dual infections and viral tropism of HIV-1 among Kenyan patients prior to widespread use of antiretroviral drugs.

**Methods:**

Remnant blood samples from consenting sexually transmitted infection (STI) patients in Nairobi were collected between February and May 2001 and stored. Polymerase chain reaction and cloning of portions of HIV-1 *gag*, *pol *and *env *genes was carried out followed by automated DNA sequencing.

**Results:**

Twenty HIV-1 positive samples (from 11 females and 9 males) were analyzed. The average age of males (32.5 years) and females (26.5 years) was significantly different (p value < 0.0001). Phylogenetic analysis revealed that 90% (18/20) were concordant HIV-1 subtypes: 12 were subtype A1; 2, A2; 3, D and 1, C. Two samples (10%) were discordant showing different subtypes in the three regions. Of 19 samples checked for co-receptor usage, 14 (73.7%) were chemokine co-receptor 5 (CCR5) variants while three (15.8%) were CXCR4 variants. Two had dual/mixed co-receptor use with X4 variants being minor population.

**Conclusion:**

HIV-1 subtype A accounted for majority of the infections. Though perceived to be a high risk population, the prevalence of recombination in this sample was low with no dual infections detected. Genotypic co-receptor analysis showed that most patients harbored viruses that are predicted to use CCR5.

## Background

The HIV/AIDS epidemic is a major global public health crisis. Currently, an estimated 33 million people worldwide are living with HIV-1 infection. The majority of cases (67%) are in sub-Saharan Africa [1,2]. Evolution of HIV-1 has assumed multiple guises which differ in geographic distribution [3]. Three groups of HIV-1 have developed across the globe: M (major), O (outlying) and N (new) [4]. Majority of HIV-1 subtypes responsible for the AIDS pandemic belong to group M and phylogenetic analysis has further classified them into 11 pure HIV-1 subtypes [5,6] and 43 circulating recombinant forms (CRFs)[7]. In Kenya, reports of diverse HIV-1 subtypes and recombinants abound [8-10]. Subtype A1 and its recombinants are the most prevalent and responsible for majority of AIDS cases [11], their presence in other regions of the world not withstanding [12].

HIV requires a co-receptor [initially chemokine (C-C motif) receptor5 (CCR5)] for entry into its host to facilitate primary infection irrespective of the transmission route and the predominant viral tropism present in the donor [13]. Most HIV variants isolated from drug-naive, chronically-infected individuals use CCR5 along with CD4 to gain entry into cells - hence referred to as R5-tropic. On the other hand viruses able to use CXCR4 co-receptors (X4-tropic) tend to emerge later over the course of HIV infection, being recognized in nearly half of patients in advanced disease stages. Under drug therapy, consequent switches back and forth between both co-receptors may occur[14].

Studies have shown that AIDS progression differs as a function of the infecting subtype and viral tropism [15,16]. Studies assessing the effect of co-receptor usage on current antiretrovirals and drug resistance mutations are particularly needed since novel compounds would be used in antiretroviral-experienced patients or in subjects with drug-resistant viruses. Furthermore, HIV diversity is important for viral load testing in clinical settings as this has an impact on virus quantification[17], especially in resource-poor settings whose populations are apparently infected with diverse subtypes.

Antiretroviral therapy (ART) using nucleoside- and non-nucleoside reverse transcriptase inhibitors (NRTIs and NNRTIs) as well as protease inhibitors (PIs) has sharply reduced HIV morbidity and mortality in developed countries but has created the problem of drug resistance. Drug resistance mutations associated with most regimens have previously been described [18-21]. The emergence of resistance has fuelled the search for new drug classes with novel mechanisms of action. Different classes of entry inhibitors have been developed as alternatives for those failing therapy. Enfuvirtide, a fusion inhibitor, was the first molecule to obtain approval [22]. The approval of Maraviroc (a CCR5 antagonist) is promising and evidence of its success has been reported [23,24]. Thus, the use of co-receptor antagonists among patients failing ART has been successful in industrialised countries, but has yet to reach patients in resource-poor countries.

The aim of this study was to determine HIV-1 subtypes, recombination and dual infections among Kenyan patients before antiretroviral therapy became widely available. We further investigated co-receptor use among common infecting subtypes for alternative therapy with envisaged treatment failure.

## Methods

### Study site, population and samples

The study was carried out at the special treatment center in Nairobi. The center has been specially established to offer subsidized health services to individuals with sexually transmitted infections (STIs). After informed consent and ethical approval from the National ethical committee through Kenya Medical Research Institute, sociodemographic data (age and gender) were obtained for each individual using a self-reporting questionnaire. Three mililiters of remnant blood samples from volunteer patients, aged 18 years and above, were collected and shipped to the laboratory. The patients were not followed up after the initial sample collection. At the laboratory, the samples were tested for HIV antibodies by ImmunocombII HIV1 & 2 Bispot (Orgenics, Israel) and confirmed using an enzyme immunosorbent assay (Enzygnost, Dade-Behring, Marburg, Germany). Confirmed positive samples were separated into plasma and lymphocytes by density gradient centrifugation. Plasma and buffy coats were stored at -80°C until use. Genomic DNA was extracted from buffy coats using DNAzol (Invitrogen, Life technologies, USA) lysis and ethanol precipitation.

### Polymerase chain reaction, Cloning and Sequencing

A portion of the HIV-1 gag gene (covering amino acid 132 of p24 to amino acid 20 of p7) was amplified as previously described [9]. Portions of the HIV-1 pol and env-C2V3 regions were amplified using gene specific primers as previously described [18].

Cloning of amplified DNA fragments was performed as described before [18]. Sequencing was done on an automated ABI 310 sequencer (Applied Biosystems, Foster city, USA). At least 5 clones from each sample were analyzed to explore the possibility of dual infections. Generated sequences were aligned with subtype reference sequences from Los Alamos database using Clustal W (version 1.83) and phylogenetic trees infered by the neighbor-joining method as described [18,25]. Sequences with premature stop codons were excluded from final analyses. Profile of trees was inferred using Tree View (version 1.6.6) [26]. Bootstrap re-sampling (1,000 data sets) of multiple alignments was performed to test the statistical robustness of the trees.

### Genotypic prediction of co-receptor usage

Genotypic predictions of co-receptor usage take the relevant parts of the viral genome - V3-loop - which is a third highly variable loop of *gp120*. Even though other parts of the genome seem to influence co-receptor usage, this has remained more reliable in resource-poor settings where phenotypic assays are expensive. Genotypic methods use simple rules, such as the 11/25 rule which predicts X4 merely on the basis of the presence of basic side chains in residues 11 or 25 of the V3-loop (35 amino acids). This method also uses the overall net charge of the 35 amino acids in the V3 loop; showing R5 viruses with a net charge of 5 or less and X4 viruses with a net charge of more than 5 [13]. Based on this, classification into CCR5 users (R5), CXCR4 users (X4) or dual/mixed tropic (R5X4) is possible. Dual classification into X4 and non-X4 is also possible and most useful as it closely reflects the clinically relevant problem [14]. To improve on sensitivity, multiple clones from each sample were analyzed.

## Results

### Study population and samples

Twenty HIV-1 positive blood samples were analyzed in this study. Of these, nine were from males and 11 from females. The age difference between males (32.5 years) and females (26.5 years) was highly significant (p value < 0.0001). Study patients' characteristics are shown in table 1.

**Table 1:** Characteristics of the STI patients in Nairobi

Variable		All (n = 20)	Females (n = 11)	Males (n = 9)
Mean age in years (Range)		29 (18-37)	26.5* (18-37)	32.5* (24-36)
HIV-1 subtype				
	A1	12	5	7
	A2	2	1	1
	C	1	-	1
	D	3	3	-
	Discordant	2	2	-

* t test p value < 0.0001

### HIV-1 Subtypes

**Figure 1:**
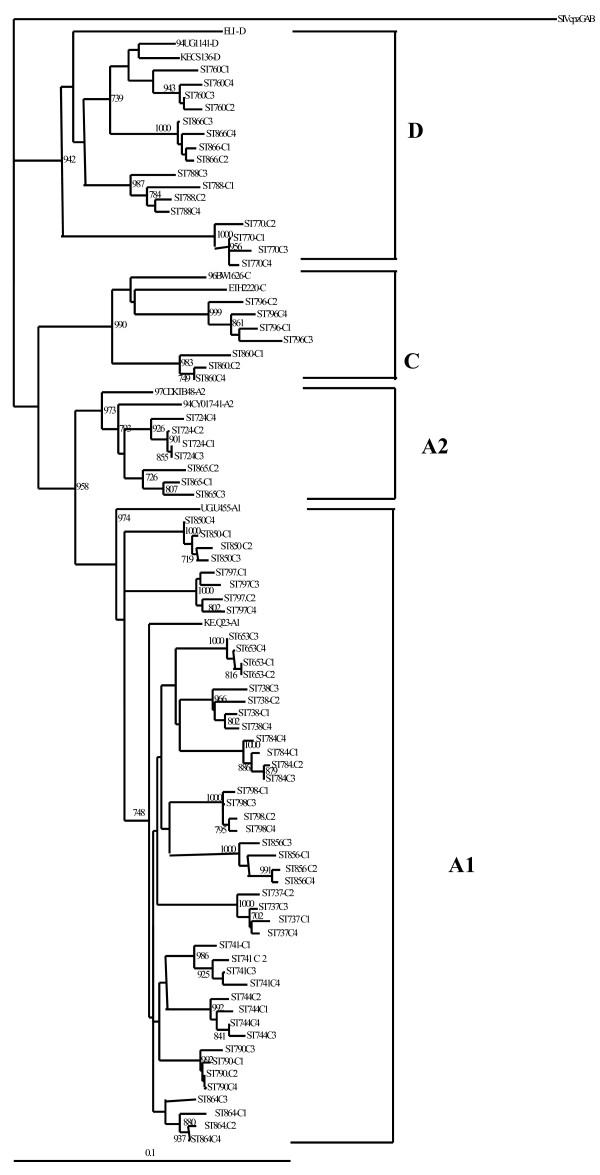
**Phylogenetic tree of HIV-1 *gag *gene**. Clones generated from each patient sample were aligned and compared with reference sequences obtained from the Los Alamos HIV database. Phylogenetic relationships were constructed by neighbor-joining method and rooted with SIVcpzGAB. The bootstrap values (of 1000 replicates) above 70% are indicated next to the node. Brackets on the right indicate the subtype clusters.

**Figure 2:**
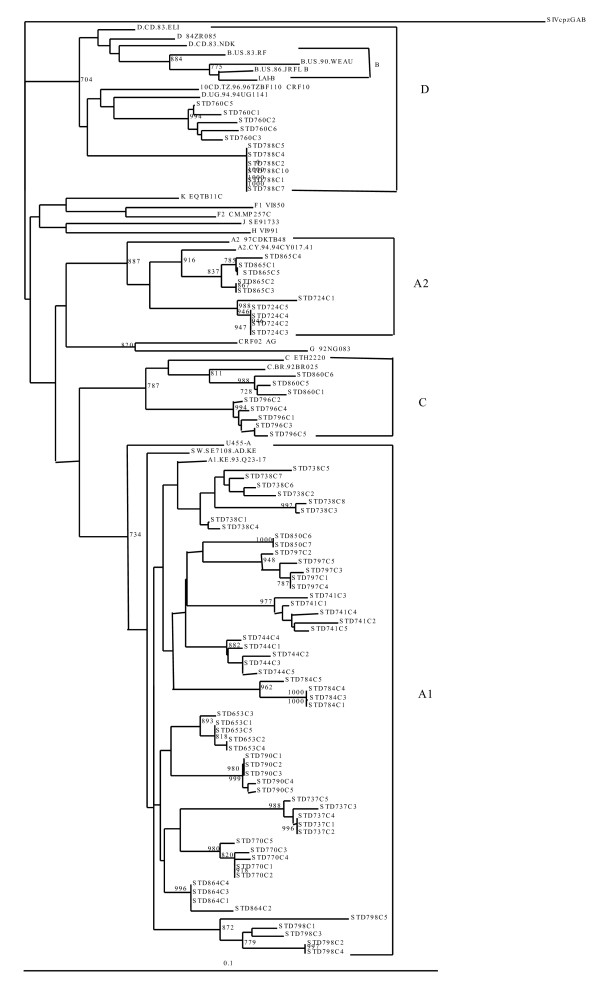
**Phylogenetic tree of HIV-1 *pol *gene**. Cloned patient samples were aligned and compared with reference sequences obtained from the Los Alamos HIV database. Phylogenetic relationships were constructed by neighbor-joining method and rooted with SIVcpzGAB. The bootstrap values (of 1000 replicates) above 70% are indicated next to the node. Brackets on the right indicate the subtype clusters.

**Figure 3:**
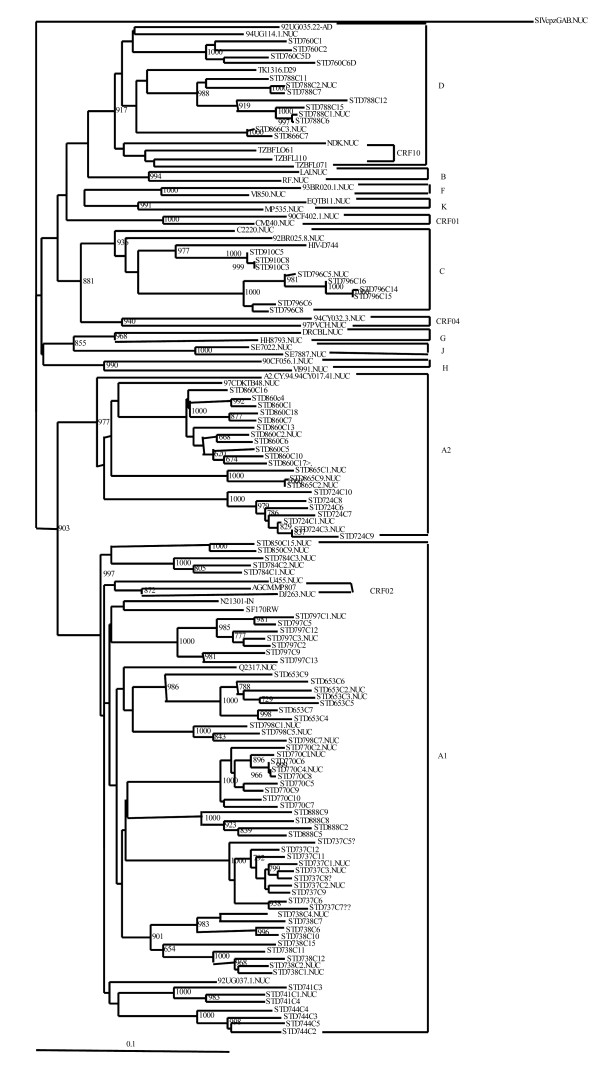
**Phylogenetic tree of HIV-1 *env-C2V3 *region**. Clones generated from each patient sample were aligned and compared with reference sequences obtained from the Los Alamos HIV database. Phylogenetic relationships were constructed by neighbor-joining method and rooted with SIVcpzGAB. The bootstrap values (of 1000 replicates) above 70% are indicated next to the node. Brackets on the right indicate the subtype clusters.

The sample sequences were assigned to subtypes if at least two gene fragments were successfully amplified. Phylogenetic analysis revealed that 18 (90%) were concordant subtypes (12 subtype A1, 2 subtype A2, 3 subtype D and 1 subtype C) and clustered together with respective reference sequences from the HIV database. However, one sample did not amplify in the *env *gene but was classified as a pure subtype A1 since it amplified in *pol *and *gag*. Sequences from two samples (10%) were discordant, showing different subtypes in the analyzed regions. No dual infections were observed. All male patients were infected with concordant subtypes (7 had subtype A1; One had subtype C and another subtype A2). The two samples with discordant subtypes (C/C/A2 and D/A/A in *gag*, *pol *and *env*, respectively) and three with subtype D were found in females' blood samples. Table 2 shows the subtypes determined in the analyzed gene fragments of each patient's virus. Phylogenetic trees for *gag*, *pol *and *env *are shown in figures 1, 2 and 3, respectivesly.

**Table 2:** HIV-1 subtypes and predicted viral tropism among STI patients in Nairobi

Sample Code	Age (years)	Gender	HIV-1 Subtype			Predicted phenotype (clones/sample)	
					
			gag	Pol	Env	R5	X4
653	25	F	A1	A1	A1	5/6	1/6
724	25	F	A2	A2	A2	5/6	1/6
737	27	F	A1	A1	A1	9/9	-
738	34	M	A1	A1	A1	9/9	-
741	35	M	A1	A1	A1	5/5	-
744	36	M	A1	A1	A1	5/5	-
760	23	F	D	D	D	-	5/5
770	29	F	D	A1	A1	9/9	-
784	35	M	A1	A1	A1	5/5	-
788	25	F	D	D	D	6/6	-
790	32	F	A1	A1	A1	4/4	-
796	31	M	C	C	C	5/5	-
797	24	M	A1	A1	A1	7/7	-
798	24	F	A1	A1	A1	5/5	-
850	32	M	A1	A1	A1	4/4	-
856	37	F	A1	A1	N/A	-	-
860	26	F	C	C	A2	10/10	-
864	33	M	A1	A1	A1	5/5	-
865	33	M	A2	A2	A2	-	4/4
866	18	F	D	D	D	-	5/5

N/A: Not amplified. F: Female. M: Male. R5: CCR5-tropic. X4: CXCR4-tropic.

### HIV-1 co-receptor usage

Nineteen samples were checked for viral tropism; one sample could not be successfully amplified and cloned in the *env *gene despite having been amplified in the other two genes. In this region 11 were subtype A1, 3 were subtype A2, 2 were subtype C and 3 were subtype D. Fourteen samples (73.7%) were R5 variants, while three were X4 variants. One sample among subtype A1 had mixed co-receptor usage. One sample among subtype A2 had X4 using virus while another had mixed co-receptor usage. Among subtype D, 2 samples had X4 and one sample had R5 variants, respectively. All subtype C sequences were R5 variants (Table 2).

## Discussion

In the current study, 20 samples were studied and found to habour diverse HIV-1 subtypes. Majority of sequences were subtype A1. This is in agreement with previous reports that have also shown continuous evolution of HIV-1 subtype A to form sub-subtypes which have been reported in different continents [8,27,28]. The increased diversity pose complexities in management of drug resistant variants. Currently, ART is being scaled up in Kenya [29]. The consequences of this upscaling is yet to be evaluated. Although we did not test for HIV drug resistance in this population, the rapid scale up of ART is expected to lead to an increase in drug resistant strains among drug-naïve patients. This is likely to result in increased prevalence of resistance.

In this study, prevalence of recombination among STI patients was comparatively low despite the fact that STI patients are considered to be a high risk population. Although we employed a commonly used subtyping methodology, the absence or presence of recombination in the three gene fragments that were analyzed does not exclude the possibility of recombination elsewhere. Furthermore, our sample size was small. Nonetheless, the possibility of recombination and emergence of newer strains is likely as infected populations move from areas with high prevalence into Nairobi in search of better living standards. From our observation of gender and age, the likelihood that our patients were primarily female prostitutes and their male customers cannot be ruled out. Since our sample population was a self selected type, it may not reflect HIV dynamics in the general population at the time of sampling. This calls for continued monitoring of circulating HIV-1 subtypes and drug resistance in the general population as treatment options become increasingly available in resource-poor settings. This will eventually help in understanding the dynamics of HIV strains locally and in the region for better management of the infected.

Co-receptor tropism of any given HIV isolate is closely associated with disease progression. HIV-1 co-receptor use in this study was predicted based on the amino acid composition of the V3 loop. Though this genotypic assay is less sensitive and need validation, it is relatively fast and less expensive especially in resource-poor settings. However, the use of this method to check for co-receptor usage might have affected the outcome. A more sensitive and reliable phenotyping assay would be ideal to elucidate this.

When using entry inhibitors in therapy, monitoring viral tropism will allow prediction of drug efficacy to avoid treatment failure [30,31]. Even in patients with a dominant non-X4 virus, minorities of X4 variants exist [32]. This means that detection of X4 minorities is of clinical value [33]. Therefore, time-saving, reliable and widely available methods for prediction of viral tropism are required. This is essential in guiding the use of entry inhibitors in resource-poor settings since patients are increasingly presenting with strains that are resistant to available NRTI, NNRTI and PIs. New studies on co-receptor use before and during antiretroviral treatment are required to address issues surrounding viral tropism in HIV variants among patients in whom conventional therapy has failed.

## Conclusion

HIV-1 diversity and viral tropism have emerged as major aspects in HIV therapy. Since different quasispecies can be present in patients, some are likely to outgrow others when subjected to non suppressive therapy leading to resistance and treatment failure. This is bound to complicate treatment options with the upscaling of ART in resource-poor settings. In Kenya, this is expected to lead to increased drug resistance among drug-naïve patients hence requirements for alternative drugs. Entry of new drugs with novel targets into the market will require that subtype and viral tropism determination be done before start of treatment. This, along with proper laboratory monitoring, will help in deciding best options for those infected with mixed viral populations at a time when more patients are presenting with HIV-1 subtypes that are resistant to available drugs.

## Competing interests

The authors declare that they have no competing interests.

## Authors' contributions

RWL conceived/designed the study, carried out the molecular genetic studies and drafted the manuscript. EMS and FAO participated in study design and coordination. EPM participated in designing the study and manuscript preparation. SAK participated in sequence alignments and manuscript preparation. RML participated in sequence alignments. NJL carried out blood separation. JKM performed serological assays. JGK did DNA extraction. All authors read and approved the final manuscript.

Sequence data

Sequences generated from this study were deposited at the gene bank under accession numbers: gag, AY706249 - AY706310; pol, AY704471-AY704549; env, AY705549-AY705652

## Pre-publication history

The pre-publication history for this paper can be accessed here:

http://www.biomedcentral.com/1471-2334/9/215/prepub
